# Evaluating Outpatient Parenteral Antimicrobial Therapy (OPAT) Clinic for Pediatric Infectious Conditions: Insights From Alberta Children’s Hospital

**DOI:** 10.1155/cjid/9586294

**Published:** 2026-04-05

**Authors:** Danah Alsharrah, Tajdin Jadavji

**Affiliations:** ^1^ Department of Pediatrics, Alberta Children’s Hospital, Cumming School of Medicine, University of Calgary, Calgary, Alberta, Canada, ucalgary.ca; ^2^ Infectious Diseases, Department of Pediatrics, Alberta Children’s Hospital, University of Calgary, Calgary, Alberta, Canada, ucalgary.ca

## Abstract

**Background:**

The Outpatient Parenteral Antimicrobial Therapy (OPAT) Clinic at the Alberta Children’s Hospital (ACH) offers a vital service, enabling stable pediatric patients to receive intravenous (IV) antimicrobial therapy on an outpatient basis. Established in 1997, the program aims to reduce hospital admissions and enhance patient experience. Eligible patients with various infectious conditions receive initial treatment in the emergency department before transitioning to outpatient care. The program not only improves patient satisfaction but also minimizes the risks associated with hospital stays, such as hospital‐acquired infections, while addressing the challenges of antibiotic stewardship and monitoring for potential complications. This study seeks to characterize OPAT outcomes in pediatric patients, filling a gap in existing literature.

**Methods:**

We conducted a retrospective descriptive study reviewing records of patients under 18 years of age treated with IV antimicrobials as outpatients within the OPAT program. Data were collected from electronic hospital records from May 1, 2023, to May 31, 2024. All data entries were recorded in REDCap as our data management platform.

**Result:**

The study included 902 pediatric participants, with 455 males (50.4%); 32.9% were 1–5 years of age, followed by 26.9% of 6–10 years of age. The most common diagnoses were cellulitis (15.7%), genitourinary (GU) infections (15.5%), dental infections (9.8%), and pneumonia (8.2%). Ceftriaxone was the primary IV antibiotic for GU infections, while cefazolin was commonly used for cellulitis and dental infections. Of the total participants, 8% required hospitalization due to worsening infection or to increase the frequency of antibiotics. Additionally, a higher rate of admission was noted in males (*p* = 0.03). Most patients (97%) received peripheral IV access with minimal complications. Overall, OPAT demonstrated high efficacy and safety in managing pediatric infections.

**Conclusion:**

This study underscores the safety of OPAT. Supported by a multidisciplinary team, the program effectively treated common infections primarily using ceftriaxone. Most patients maintained peripheral IV access without significant complications. While hospitalization rates were low, notable differences between male and female patients warrant further investigation into contributing factors to these disparities.

## 1. Introduction

The Outpatient Parenteral Antimicrobial Therapy (OPAT) Program at the Alberta Children’s Hospital (ACH) provides a unique opportunity for stable pediatric patients to receive their intravenous (IV) antimicrobial therapy as outpatients on a daily basis while remaining at home with their families and avoiding hospital admission. The implementation of OPAT for both pediatric and adult populations has been described worldwide as a quality improvement initiative that is safe and effective in managing various infectious conditions.

ACH has approximately 300 beds, providing a range of specialized services for pediatric patients. This capacity allows the hospital to accommodate a diverse array of medical needs, from routine care to complex treatments for various health conditions. The OPAT clinic established in 1997 was born as a result of a collaboration between the pediatric infectious disease team, emergency department (ED), pharmacy, ACH administration, and allied healthcare providers such as registered nurses and child life specialists, aiming to reduce hospital admissions and improve the overall healthcare experience for pediatric patients.

The OPAT process incorporates direct referrals from the ED for patients with infectious conditions who are clinically stable and do not require additional supportive interventions, such as supplemental oxygen or IV fluids, but require IV therapy or intramuscular therapy if an IV is not established. This service is not associated with any cost to the patients or parents. All OPAT patients are assessed daily in the clinic, which operates year‐round, including weekends and holidays (except December 25, 26, and January 1, when follow‐up is provided through the ED to ensure continuity of care). Most OPAT services described in the literature and established in hospitals provide mainly home‐based services. However, in our center we provide two separate programs, one being the OPAT clinic, where cases are referred from the ED for a short IV antibiotic course, and the other one is the Home Parenteral Therapy Program (HPTP), where most patients are referred from the inpatient units with a peripherally inserted central catheter (PICC) requiring prolonged IV antibiotics for more than 7 days.

**Figure 1:**
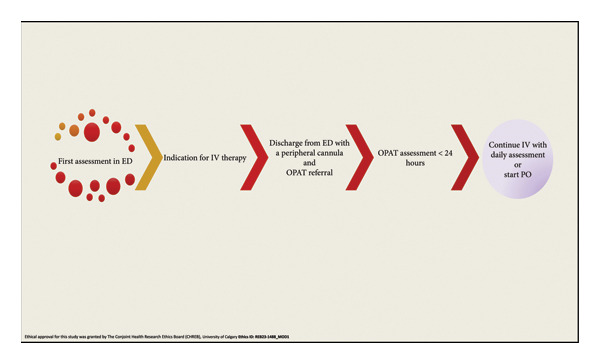
Patient’s referral process from ED to OPAT.

Indications for OPAT eligibility include infections such as cellulitis, pneumonia, urinary tract infections, fever in returning travelers pending culture results, and febrile infants pending cultures—all of which may necessitate a short term (defined in our program as less than 7 days) of IV antibiotics before transitioning to an oral regimen. Eligible patients receive their initial IV antibiotic dose in the ED and are subsequently discharged home with a peripheral cannula in place, pending assessment in the OPAT clinic within 24 hours. Upon evaluation in the OPAT by an infectious disease specialist, the physician determines whether the patient requires continued IV antimicrobial therapy based on clinical status and diagnostic findings. This assessment will dictate whether the patient remains in the OPAT for daily evaluations or is discharged home with or without an oral antibiotic regimen (Figure 1).

The implementation of OPAT has been proven in many studies to increase patient satisfaction and has been described to be safe and effective [1, 2]. There are many other benefits of avoiding hospital admissions, including reducing the risk of nosocomial infections and reducing costs [1, 2]. Some of the complications that are monitored and could be encountered in an OPAT program include overuse of IV broad‐spectrum antibiotics, subsequent hospital admission and failure of OPAT therapy, line‐associated complications, and side effects of antimicrobial therapy [3, 4]. Therefore, OPAT as a quality improvement initiative should be meeting certain standards of care, such as evidence‐based antibiotic prescription patterns and avoiding delays in care and admission, and the short‐term nature of the OPAT is adhered to without any added burden for the family. In addition, there has been a paucity of studies on the outcomes of OPAT in pediatrics. This study helps us characterize the types of infections that we treat and define the outcomes and complications that could be encountered.

## 2. Methods

We conducted a retrospective descriptive study reviewing electronic medical records of patients under 18 years of age, treated with IV antimicrobials as outpatients within the OPAT program at ACH. Data collected were between May 1, 2023, and May 31, 2024. All data entries were systematically recorded in REDCap, a data management platform.

### 2.1. Statistical Analysis

We employed Chi‐square tests to analyze categorical variables and assess associations between groups. The Chi‐square test was utilized to evaluate the relationships between variables such as sex, clinical diagnosis, and health outcomes, including hospital admissions, with a significance level set at *p* < 0.05. In cases where expected frequencies were low, Fisher’s exact test provided a more appropriate alternative to ensure the validity of our results. In our statistical analysis, we chose not to correct Type I error, as this was an exploratory analysis aimed at identifying potential associations.

### 2.2. Ethical Approval

Ethical approval for this study was granted by the Conjoint Health Research Ethics Board (CHREB), University of Calgary. The study was conducted in accordance with ethical standards, ensuring the confidentiality and protection of patient data. Due to the retrospective design, informed consent was waived, and all data were anonymized to safeguard participant identities. Ethics ID: REB23‐1488_MOD1.

## 3. Results

A total of 902 participants were included, with 455 males (50.4%) and 447 females (49.6%). Age distribution revealed 297 (32.9%) in the 1–5 years age group and is detailed in Table 1.

**TABLE 1:** Demographic data, comorbidities, and referral source.

Sex	Male	455 (50.4%)
	Female	447 (49.65%)

Age	< 3 months	28 (3.1%)
	3–6 months	14 (1.6%)
	6 months–1 year	52 (5.8%)
	1–5 years	297 (32.9%)
	6–10 years	243 (26.9%)
	11–15 years	148 (16.4%)
	16–18 years	120 (13.3%)

Comorbidities	Yes	230 (25.5%)
	No	672 (74.5%)

Type of comorbidities	Asthma	31
	Urological	57
	Eczema	23
	Diabetes	2
	Cardiac	14
	Immunodeficiency	15
	Malignancy	4
	Neurological	24
	Sickle cell	37

Referring source	ED of children’s hospital	797 (88.4%)
	Other ED	103 (11.4%)
	Community	0

Patient seen once in APTP	Yes	471 (52.2%)
	No	431 (47.8%)

The most prevalent diagnoses were genitourinary (GU) infections in 140 patients (15.5%), followed by cellulitis (143, 15.7%), dental infections (88, 9.8%), and pneumonia (74, 8.2%). Other notable conditions included lymphadenitis (69, 7.6%), peritonsillar abscess (49, 5.4%), otitis media (49, 5.1%), and tonsillopharyngitis (43, 4.8%). Collectively, ears, nose, and throat (ENT) presentations represented the most common system involvement, accounting for a total of 253 patients, which constitutes 28% of all cases (Table 2).

**TABLE 2:** Types of infections and antibiotics used.

Type of infection	*N* (%)	Average duration of IV	Type of IV antibiotics *N* (%) None = no IV required	Type of PO
Genitourinary infections	140 (15.5%)	3.3 days	Ceftriaxone 81 (58%) Gentamicin 50 (35%) None 4	Cefixime 55 (39%) Septra 32 (23%)

Nonfacial cellulitis	117 (12.9%)	2.4 days	Cefazolin ± prob 86 (74%) Ceftriaxone 14 (12%) None 8	Cephalexin 81 (69%) Septra 12 (10%)

Facial cellulitis	26 (2.8%)	1.6 days	Cefazolin ± prob 12 (46%) Ceftriaxone 12 (46%) None 2	Cephalexin 10 (38%) Amoxiclav 11 (42%)

Dental infections	88 (9.7%)	2.5 days	Cefazolin ± prob 40 (45%) Ceftriaxone 35 (40%) Metronidazole 55 (62.5%) None 11	Amoxiclav 67 (76%) Amoxicillin 11 (12.5) Metronidazole 5 (5.6%)

Pneumonia	74 (8.2%)	2.4 days	Ceftriaxone 59 (80%) None 13	Amoxicillin 35 (47%) Amoxiclav 12 (16%)

Lymphadenitis	69 (7.6%)	2.5 days	Cefazolin ± Prob 34 (49%) Ceftriaxone 27 (39%) None 9	Cephalexin 39 (56%) Amoxiclav 10 (14.4%)

Skin abscess	61 (6.7%)	2.5 days	Cefazolin ± Prob 39 (64%) Ceftriaxone 10 (16%) None 8	Cephalexin 25 (41%) Septra 13 (21%)

Peritonsillar abscess	49 (5.4%)	3 days	Ceftriaxone 41 (83%) Cefazolin ± Prob 7 (14%) Metronidazole 28 (57%) None 3	Amoxiclav 35 (71%) Amoxicillin 7 (14.2%)

Otitis media	46 (5%)	2.8 days	Ceftriaxone 43 (93%) None 3	Amoxiclav 12 (26%) Amoxicillin 7 (15%)

Tonsillarpharyngitis	43 (4.7%)	2.1 days	Ceftriaxone 29 (67%) Cefazolin ± Prob 7 (16%) Metronidazole 6 (14%) None 7	Amoxiclav 7 (16%) Amoxicillin 15 (35%)

Periorbital cellulitis	34 (3.7%)	2.3 days	Ceftriaxone 21 (62%%) Cefazolin ± Prob 8 (23%) None 5	Cephalexin 13 (38%) Amoxiclav 13 (38%)

Sinusitis	21 (2.3%)	2.2 days	Ceftriaxone 18 (86%) None 3 (14%) None 3	Amoxiclav 15 (71%) Levofloxacin 3 (14%)
Febrile neutropenia	21 (2.3%)	2 days	Ceftriaxone 15 (71%) Cefazolin 1 (4.7%) None 5 (23%)	Amoxicillin 1 (4.7%) Cephalexin 1 (4.7%)

Parotitis	19 (2.1%)	1.7 days	Ceftriaxone 7 (38%) Cefazolin ± Prob 6 (31%) None 6 (31%)	Cephalexin 10 (52%) Amoxiclav 4 (21%)

Mastoiditis	13 (1.4%)	3.6 days	Ceftriaxone 12 (92%) None 1 (7.6%)	Amoxiclav 9 (69%) Amoxicillin 1 (7.6%)

Bacteremia	12 (1.3%)	5.1 days	Ceftriaxone 9 (75%) Cefazolin 1 Gentamicin 1 None 1	Amoxicillin 1 Azithromycin 1 Septra 1

Traveler’s fever	9 (1%)	4.6 days	Ceftriaxone 8 (89%) Gentamicin 1 (11%)	Azithromycin 2 (22%) Septra 1 (11%)

Gastroenteritis	7 (0.7%)	2.1 days	Ceftriaxone 6 (85%) None 1	Azithromycin 1 None 6 (85%)

Typhoid fever	6 (0.6%)	3	Ceftriaxone 6	Azithromycin 3 Septra 2 None 1

Retropharyngeal abscess	6 (0.6%)	3 days	Cefazolin ± Prob 4 Ceftriaxone 2	Amoxiclav 1 Cephalexin 1

Animal bite	6 (0.6%)	2.3 days	Ceftriaxone 5 Metronidazole 4 None 1	Amoxiclav 5 Moxifloxacin 1

MSK infection	5 (0.5%)	3 days	Cefazolin ± Prob 3 Ceftriaxone 1 Amoxiclav 1	Clindamycin 1 Levofloxacin 1

Orbital cellulitis	2 (0.2%)	2 days	Ceftriaxone 2	None

Viral infection	126 (14%)	2 days	Ceftriaxone 85 Cefazolin ± prob 3 None 36	Amoxiclav 3 Cephalexin 2

*Note:* PO, oral; Septra, trimethoprim and sulfamethoxazole; Prob, probenecid.

Abbreviation: IV, intravenous route.

For GU infections, the most used IV antibiotic was ceftriaxone in 81 cases and gentamicin in 50 cases. Both choices of antibiotics are considered first‐line empirical treatment options. Other antibiotics utilized included ertapenem for cultures that yield resistant bacteria. Among the urine cultures, *Escherichia coli* was the most common pathogen in which 121 patients tested positive, with non‐ESBL (extended‐spectrum beta‐lactamase) *E. coli* detected in 63 patients and ESBL‐producing *E. coli* identified in 28 patients. For cellulitis, the most used IV antibiotic was cefazolin in 98 patients, followed by ceftriaxone, with the most common oral stepdown being cephalexin. As for dental infections, the most used IV antibiotic was cefazolin in 40 cases, and ceftriaxone was started on 35 patients. Of those patients with dental infections, 62.5% required additional anaerobic coverage with metronidazole. Regarding comorbidities, 230 participants (25.5%) reported the presence of one or more comorbid conditions (Table 1).

Out of the total patients, 73 (8%) required subsequent hospitalization either due to worsening infection or for more frequent monitoring and more frequent antibiotic dosage. Notably, hospital admissions were more significant among males, with 46 (5%) needing admission, compared to 27 (3%) of females *p*‐value of 0.03. Also, 49 (5.4%) had a subsequent ED visit within two weeks. 875 (97%) of patients received peripheral IV access only. Five (0.6%) patients had previously placed PICCs, and 22 (2.4%) had no IV access in which the intramuscular route was used. Regarding the frequency of peripheral line replacement, 859 (95.2%) required no replacements, 41 (4.5%) required replacement once, and 2 (0.2%) twice. As for peripheral line‐related complications, 869 (96.3%) patients had no complications, while 5 (0.6%) experienced occlusions, 15 (1.7%) had leakage, and 11 (1.2%) had their lines pulled out. Overall, these results suggest that peripheral IV catheter use is generally safe and well‐tolerated in short‐term use, with complications occurring in only a small percentage of those patients. Two of the patients who were initially presented with lymphadenitis were hospitalized and diagnosed with malignancy. Among the patients, only 7 (0.8%) were lost to follow‐up, and there were no deaths (Table 3). In our analysis of sex and age as predictors for the five most common infections, we found no significant association between these demographic variables and subsequent hospital admissions or ED visits with insignificant *p* values (Table 4).

**TABLE 3:** Type of complications encountered with APTP service.

Patient requiring subsequent hospital	Yes 73 (8%)
	Male 46 (5%)
	Females 27 (3%) *p* value 0.03
	< 1 year 11 (94) (15%)
	1–5 years 26 (297) (35%)
	6–10 years 16 (243) (22%)
	11–15 years 14 (148) (19%)
	16–18 years 6 (120) (8%) *p* value 0.345
	No 829 (92%)

Subsequent ED visit in less than 2 weeks	Yes 49 (5.4%)
	No 853 (94.6%)

Type of IV access	Peripheral 875 (97%)
	Central 5 (0.6%)
	None 22 (2.4%)

Frequency of peripheral line replacement	None 859 (95.2%)
	Once 41 (4.5%)
	Twice 2 (0.2%)

Line‐related complications	None 869 (96.3)
	Occlusion 5 (0.6%)
	Leakage 15 (1.7%)
	Pulled out 11 (1.2%)

Patient who lost follow‐up in OPAT	7 (0.8%)

Mortality	None

**TABLE 4:** Most common infections with sex and age as a variable.

	**Number of subsequent hospital admissions (within 2 weeks)**	**p** **value**	**Subsequent ER visit *N* (total)**	**p** **value**

Genitourinary infections (140)	**Total 6**		**Total 9**	
	Female 2 (97) and Male = 4 (53)	0.76	Female 6 (97) and Male 3 (53)	0.86
	< 1 year = 4 (33), 1–5 years = 0 (29), 6–10 years = 1 (40), > 11 years = 1 (38)	—	< 1 year = 0 (33), 1–5 years = 1 (29), 6–10 years = 5 (40), > 11 years = 2 (38)	—

Cellulitis (143)	**Total 8**		**Total 6**	
	Female 4 (65) and Male 4 (78)	0.79	Female 3 (65) and Male 3 (78)	0.819
	< 1 year = 2 (11), 1–5 years = 0 (31), 6–10 years = 2 (44), > 11 years = 3 (57)	—	< 1 year = 0 (33), 1–5 years = 1 (29), 6–10 years = 5 (40), > 11 years = 2 (38)	—

Dental infections (88)	**Total 3**		**Total 3**	
	Female 1 (39) and Male 2 (49)	0.69	Female 1 (39) and Male 2 (49)	0.69
	< 1 year = 0 (0), 1–5 years = 1 (28), 6–10 years = 2 (34), > 11 years = 0 (26)	—	< 1 year = 0 (0), 1–5 years = 1 (28), 6–10 years = 1 (34), > 11 years = 1 (26)	—

Pneumonia (74)	**Total 8**		**Total 3**	
	Female 2 (33) and Male 6 (41)	0.23	Female 1 (33) and Male 2 (41)	0.68
	< 1 year = 1 (4), 1–5 years = 4 (37), 6–10 years = 1 (23), > 11 years = 1 (10)	0.59	< 1 year = 1 (4), 1–5 years = 2 (37), 6–10 years = 0 (23), > 11 years = 0 (10)	—

Lymphadenitis (69)	**Total 15**		**Total 6**	
	Female 5 (27) and Male 10 (42)	0.63	Female 2 (27) and Male 4 (42)	0.76
	< 1 year = 1 (5), 1–5 years = 9 (27), 6–10 years = 3 (25), > 11 years = 2 (12)	0.29	< 1 year = 0 (5), 1–5 years = 3 (27), 6–10 years = 3 (25), > 11 years = 2 (12)	—

## 4. Discussion

Outpatient service plays a vital role in managing patients in the healthcare setting. The utilization of OPAT was first described in the United States for cystic fibrosis patients who required prolonged IV antibiotic therapy [5]. Since then, OPAT grew substantially. The OPAT service provides IV antibiotics in a dedicated environment where they are assessed in a specialized outpatient clinic that is managed by a multidisciplinary team of pediatric infectious disease specialists, nurses, and pharmacists. Patients are monitored daily until discharged with a clear plan while ensuring patients and parents are provided with valuable resources for education. Such service can reduce the need for hospitalization and reduce the burden on the healthcare facility and possibly reduce costs [6]. In addition, several previously published studies have demonstrated patients’ and parents’ satisfaction in avoiding hospital admission [7].

The indications for administering IV antibiotics before starting an oral course vary, and determining factors include type of infection, severity, age of patient, presence of comorbidity, the availability of oral and IV agents, and many others. Overall, administering IV antibiotics is often a vital initial step in managing various infectious conditions and ensuring that patients receive appropriate and timely care tailored to their specific clinical need, which is a primary goal of OPAT. In addition, maintaining an effective narrow spectrum agent used for a specific indication is the primary goal for antibiotic stewardship [8]. Therefore, OPAT should promote rational use of IV antibiotics and promote oral switches when feasible. In this descriptive study we demonstrate the type of infections requiring assessment by an infectious disease specialist to decide on choices of antibiotics and the clinical outcomes.

We had a total of 902 patients younger than 18 years of age with data collected over a 13‐month period. One of the most prevalent infections observed was GU infections, which are a frequently encountered presentation in the pediatric ED. Guidelines suggest the use of ceftriaxone or gentamicin as first‐line empirical IV agents [9]. Although a Cochrane review found no difference between IV and PO for pediatric patients with UTI, indications for IV would include underlying urological anomaly, oral intolerability, infants less than 3 months of age, social circumstances, and ill‐looking children [10]. Therefore, some experts recommend initial IV followed by PO antibiotics when the clinical condition improves and the results of the urine cultures are back to tailor the oral therapy [11]. Other potential indications include limited availability of effective oral agents due to resistance or a high risk of resistance. However, this study did not collect detailed data on the specific reasons for choosing IV therapy. Interestingly, 30% of *E. coli* isolated in our study was ESBL positive where we have limited IV and oral antibiotics. The other commonly encountered condition was cellulitis, in which we had a total of 143 cases of combined body and facial cellulitis. The most commonly used IV antibiotic was cefazolin, mostly as a single dose combined with probenecid (70), followed by IV ceftriaxone (24), both of which are suitable first‐line agents. A randomized, double‐blind trial demonstrated that a once‐daily regimen of cefazolin plus probenecid versus ceftriaxone in moderate to severe cellulitis showed similar cure rates and safety profiles [12]. Even though most mild cases of cellulitis can be managed with oral antibiotics, some might require IV treatment specifically for moderate or severe cases or when oral therapy fails [13].

The third most common condition was dental infections. The need for IV versus oral antibiotics for dental infections is primarily guided by the severity of the infection and the patient’s overall health status. Evidence suggests that for localized infections, such as uncomplicated dental abscesses, oral antibiotics are often sufficient and effective. However, in cases of more severe infections, such as those involving deep tissue or systemic spread, IV antibiotics may be necessary alongside a surgical intervention to achieve rapid therapeutic levels and ensure adequate tissue penetration [14]. The most commonly prescribed empiric therapy for dental infections would include amoxicillin–clavulanic acid or amoxicillin with metronidazole [15]. Amoxicillin–clavulanic acid is a suitable agent, as most anaerobic bacteria cultured from dental infections are susceptible [16]. In our population, there was a noted difference in the empiric regimen for dental infections, which included ceftriaxone or cefazolin with or without metronidazole. In addition, 53% of patients with dental infections were discharged home on oral antibiotics without a second dose of an IV agent. Importantly, our data lacks the documentation of the severity of the dental infections in order to justify the need for IV antibiotics in 47% of our patients, and future studies need to address this. Other important infections that were treated in the OPAT clinic included typhoid fever, febrile neutropenia, musculoskeletal infections, bacteremia, and animal bites, some of which required daily assessment and monitoring to define duration of therapy.

Overall, ceftriaxone was the most common empirical IV antibiotic used in our OPAT clinic in 60% of patients, as it has a broad spectrum of activity to include both Gram‐positives and Gram‐negatives with a long half‐life permitting a once‐daily dose [17]. However, in certain conditions such as pneumonia, ampicillin would be an appropriate first‐line agent, but because of the frequency of 8‐hourly administration, it is not a convenient IV option for OPAT. Therefore, in these circumstances we are using a broader agent compared to a more targeted treatment due to practicality.

Interestingly, even though we have almost an equal sex distribution between males and females, it was noted that males were more likely to be hospitalized compared to females, 46 (5%) versus 27 (3%), respectively. This disparity may reflect underlying biological, behavioral, or social factors that warrant further investigation [18, 19]. Understanding these gender differences could inform tailored approaches to treatment and follow‐up care. Additionally, the data indicated that 49 (5.4%) patients had a subsequent ED visit within two weeks of treatment. This relatively low rate of emergency visits suggests that the majority of patients responded well to outpatient management, reinforcing the effectiveness of the ambulatory model for treating pediatric infections. Future studies should investigate the reasons for readmission and emergency visits, aiming to identify risk factors that could be addressed proactively to improve patient outcomes. Furthermore, implementing targeted follow‐up protocols for at‐risk populations could enhance the overall efficacy of outpatient treatment strategies.

The results of this study provide valuable insights into the use and safety of peripheral IV access in a pediatric outpatient setting. The data on the frequency of peripheral IV line replacement are particularly encouraging, with 95.2% experiencing no line replacements during the treatment course. Complications related to IV access were infrequent. The low rate of complications in this cohort supports previously published data on the safety of OPAT. These findings indicate that short‐term use of peripheral IV catheters has a favorable safety profile when properly managed, particularly in an outpatient setting where continuous monitoring may be more challenging.

This study has several limitations, including being a retrospective analysis, lack of a detailed analysis of patient characteristics, and variations in treatment protocols. Furthermore, the study did not capture patient‐reported outcomes, such as quality of life or patient satisfaction, which are important indicators of the effectiveness and acceptability of outpatient IV therapy. This study is further limited by the absence of clearly defined criteria guiding the selection of IV over oral antibiotic therapy. The high number of OPAT cases identified raises the possibility that some patients may have been appropriate candidates for initial oral therapy or for an earlier transition from IV to oral treatment.

Future studies in the context of outpatient IV antibiotic therapy should explore several key areas to enhance patient outcomes and refine treatment protocols. In conclusion, this study highlights the critical role of utilizing (IV) antibiotics through the OPAT clinic. The service effectively provides IV antibiotics in a specialized outpatient environment, supported by a multidisciplinary team of pediatric infectious disease specialists, nurses, and pharmacists.

## Funding

No funding was received for this manuscript.

## Conflicts of Interest

The authors declare no conflicts of interest.

## Data Availability

The data that support the findings of this study are available on request from the corresponding author. The data are not publicly available due to privacy or ethical restrictions.
